# When is a muscle at rest? The problem of background activity in brain stimulation

**DOI:** 10.1113/EP094122

**Published:** 2026-07-23

**Authors:** Nicholas P. Holmes

**Affiliations:** ^1^ School of Sport, Exercise and Rehabilitation Sciences University of Birmingham Birmingham UK

**Keywords:** electromyography, muscle, transcranial magnetic stimulation

“Relax. Don't move! Just stay completely still while I repeatedly electrocute your head.”

These are not the threats of a medieval torturer, but the instructions of a brain stimulation scientist to their volunteers. For 41 years, researchers using transcranial magnetic stimulation (TMS) have applied brief, usually painless, electromagnetic pulses to human heads. They have done this not just for fun, but primarily to understand how the brain generates and controls the body's movements. They've done this in thousands of studies, often evoking and measuring responses in the small muscles of the hand. Critically, they have done this mostly while the muscles are at rest.

But when is a muscle at rest, ask O'Sullivan and colleagues in this issue of *Experimental Physiology* (O'Sullivan et al., 2026). A common answer to this question involves recording electrical activity from the muscle (‘electromyography’), and setting an arbitrary voltage below which we decide that the muscle is ‘at rest’. This cut‐off voltage is sometimes given as a range between the minimum and maximum over a certain period (e.g., ‘less than 50 µV over 200 ms’), and other times as a deviation from the mean signal (‘the square root of the mean squared voltage, RMS, is less than 10 µV over 200 ms’). This cut‐off can be applied during data collection itself, so that measures are not taken, or are re‐taken, if the muscle was not at rest. More commonly it is done ‘offline’ as a pre‐processing step.

O'Sullivan and colleagues focus on the optimal pre‐processing cut‐off to decide whether a muscle is at rest or active. They note that this cut‐off varies dramatically in previous research, from 2.5 µV in some studies, to 20 µV in others, and as high as 100 µV in others. This 40‐fold range in the cut‐off criterion used across studies implies that in TMS research, some resting muscles are more at rest than others.

These differences in methods might not matter so much if the brain controlled the muscles in a simple linear manner, if everyone's muscles were the same, and if researchers measured muscle activity in the same way. Unfortunately, none of these conditions apply. The brain activates the hand muscles in non‐linear and dynamic ways as hand movements evolve from plan to execution and control, even during everyday movements such as reach and grasp (Holmes et al., 2024; Lemon et al., 1995), and there is considerable natural variation between people, muscles and methods (Besomi et al., 2024).

Given the wide variation in existing practice, O'Sullivan and colleagues examined how different levels of background activity predict the likelihood of muscle responses to TMS. A brief pulse of TMS over the motor cortex can produce a brief muscle response. Decrease the TMS intensity and the response decreases. Keep decreasing TMS intensity until half the time the muscle responds, and half the time it does not, and you have found the resting motor threshold. Now the crucial bit: does the background activity in resting muscles predict the probability of responses to TMS at threshold?

Yes. Figure 2 in their paper shows, first, that there is a continuous relationship between background muscle activity and the likelihood of response to TMS, second, that background activity as low as 1 or 2 µV predicts the response to TMS above chance, and third, that this relationship is similar in healthy participants (*n* = 45) and in those with amyotrophic lateral sclerosis (ALS, *n* = 35).

These results are problematic. Has 41 years of TMS research on resting muscles not, in fact, been done on resting muscles? Has it instead been done on muscles in a wide range of activation states, from ‘at complete rest’ to ‘rather twitchy indeed’? Which parts of our understanding of TMS and electromyography methods and – more importantly – of the brain's control of movement, need updating?

The results of O'Sullivan and colleagues’ work have wide implications for TMS research in general. One response to their challenge could be for researchers to set their background activity cut‐offs so low – for example at 1 or 2 µV – that they are certain only to record ‘resting’ muscles. But hardware quality, electrical activity and noise levels differ between labs, muscles, physiological states and people. Most importantly, lowering the cut‐off would exclude participants – for example those with ALS – who cannot voluntarily rest their muscles to the same degree as others. An alternative response is that we account for background muscle activity by including it as a variable in our statistical models of corticospinal responses (Carson, 2026).

I favour the second response. There may be no absolute level of muscle activity below which our data will be unaffected by ongoing processes in the neuromuscular system. We must not, therefore, exclude any data on arbitrary criteria; instead we must routinely measure and model the covariates that matter. This will improve the fit between data and theory, and our empirical inferences will move – ever so slightly – closer to the answers we seek.

Beyond raising questions about specific numerical cut‐offs used in the analysis of electromyography data, we should take this opportunity to ask: Why are we studying the brain's control of movement in resting muscles *at all*? The brain has a motor cortex not just to suppress, but to generate and control movement. By embracing the fact that the brain and body are never truly at rest, we can free ourselves from the arbitrary cut‐offs and thresholds that have dominated the first 41 years of TMS research (10 µV, 50 µV, 1 mV, *ad nauseum*…). Instead, I look forward to a new era of studying the dynamic non‐linearities of the corticospinal system during natural movement.

Don't relax. Move!

## AUTHOR CONTRIBUTIONS

Sole author.

## CONFLICT OF INTEREST

None declared.

## FUNDING INFORMATION

None.

## GENERATIVE AI STATEMENT

No generative AI was used in the preparation of the manuscript.
